# The mitochondrial genome of *Syneches medoganus* (Diptera: Empididae)

**DOI:** 10.1080/23802359.2021.1906177

**Published:** 2021-04-09

**Authors:** Wei Zeng, Yan Gu, Ding Yang

**Affiliations:** ^a^College of Plant Protection, China Agricultural University, Beijing, PR China

**Keywords:** Mitochondrial genome, Empididae, *Syneches*, phylogeny

## Abstract

The mitogenome of one dance fly species, *Syneches medoganus*, was sequenced as the new representative of subfamily Hybotinae. The nearly complete mitogenome was 16128 bp totally, consisting of 13 protein-coding genes (PCGs), two *rRNA* genes, and 22 *tRNA* genes. The nucleotide composition biased toward A and T, and the overall A + T was 78.1% of the entirety. All the PCGs started with codon ATT, ATC or ATG, except CO1 using TCG as an initiation codon. Most PCGs terminated with stop codon TAA or TAG except CO2 with an incomplete stop codon T. Bayesian analyses indicated that the monophyly of Hybotinae subfamily group is strongly supported. Subfamily Hybotinae is the sister group of subfamily Tachydromiinae.

## Introduction

Empididae is one of the largest groups in Brachycera (Diptera). They can be easily identified by the small round head and humpbacked thorax (Yang et al. 2007). The genus *Syneches* is a large genus in the subfamily Hybotinae with over 140 known species worldwide (Wang et al. 2014).

The adult specimens of *Syneches medoganus* used in this study were collected in Motuo of Tibet (95°13′52′′N, 29°19’28′′E, 1950 m) by Qicheng Yang and identified by Ding Yang. Specimens are deposited in the Entomological Museum of China Agricultural University (CAU), Beijing (Voucher number: CAU-YDEMPI-Para-1). The genomic DNA was extracted from the muscle tissue of specimens following the instruction of DNeasy DNA Extraction kit (TIANGEN, Beijing, China) and stored at −20 °C refrigerator. DNA samples were pooled for next-generation sequencing library construction. The quantified DNA samples were included in a single pool, and the pooled dsDNA sample was sent to BerryGenomics CO., LTD for library construction and sequenced by the Illumina NovaSeq6000. The final filtered reads were assembled with the de novo assembler IDBA_UD (Peng et al. 2012). A similarity threshold of 98% and minimum and maximum k values of 80 and 240 bp were used for assemblies. The decoy sequence COI was amplified by standard PCR, and BLAST search was performed with BioEdit version 7.0.5.3 (https://file.org/free-download/bioedit). The Björn Canbäck Bioinformatics Online services (http://130.235.244.92/ARWEN/) was used to identify the position of all tRNA (Laslett and Canbäck 2008).

**Figure 1.**
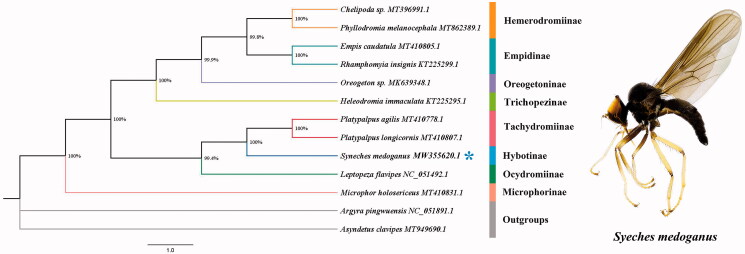
The phylogenetic tree of BI analysis based on 13 PCGs and adult of *Syneches medoganus* Zhao, Ding, Lin, & Yang, 2020. ‘*’ Indicated new sequenced data in this study.

The almost complete mitogenome of *Syneches medoganus* was 16,128 bp totally, which consisted of 13 protein-coding genes (PCGs), 22 transfer *RNA* genes (tRNAs), and two *rRNA* genes (rRNAs). The gene arrangement and gene lengths are conventional with other dipteran flies reported earlier (Day et al. 2016; Karagozlu et al. 2017; Li et al. 2019; Chen et al. 2020). The nucleotide composition was 39.2% of A, 38.9% of T, 8.7% of G, and 13.2% of C. The ATT and ATC were start codon shared with ND2, ATP8, ND3, ND5, ND6, ND1, and start codon ATG was shared with CO2, ATP6, CO3, ND4, ND4L, and COB. Particularly, CO2 initiated with codon ATG. The conservative stop codon TAA and TAG were shared with most of the PCGs except for one gene, CO2 terminated with an incomplete stop codon T. Together with mitogenome data of other 12 Diptera species retrieved from NCBI (the Accession Numbers are indicated in Figure 1), we conducted phylogenetic analysis based on 13 PCGs. The phylogeny was reconstructed with Bayesian inference (BI) under GTR model in MrBayes version 3.2.7a (http://nbisweden.github.io/MrBayes/) (Ronquist et al. 2012). The topology and nodal support value are shown in Figure 1. BI analysis indicated that Microphorinae is the basal group of Empididae. The rest seven subfamilies form two main clades: one clade of Empidinae subfamily group (Clinocerinae + (Oreogetoninae + (Hemerodromiinae + Emipidinae))) and another clade of Hytotinae subfamily group (((Tachydromiinae + Hybotinae) + Ocydromiinae). The monophyly of Hybotinae subfamily group is strongly supported. Subfamily Hybotinae is the sister group of subfamily Tachydromiinae. The mitogenome of *Syneches medoganus* could provide important information for the further studies of Empididae phylogeny.

## Data Availability

The data that support the findings of this study are openly available in [NCBI] at [https://www.ncbi.nlm.nih.gov/], reference number [MW355620.1].
